# Risk adapted dose-intensified postoperative radiation therapy in prostate cancer patients using a simultaneous integrated boost technique applied with helical Tomotherapy

**DOI:** 10.1186/s13014-017-0862-4

**Published:** 2017-08-10

**Authors:** Marcus Beck, Peter Wust, Tomasz Barelkowski, David Kaul, Alexander-Henry Thieme, Sascha Wecker, Waldemar Wlodarczyk, Volker Budach, Pirus Ghadjar

**Affiliations:** Department of Radiation Oncology, Charité Universitätsmedizin Berlin, Augustenburger Platz 1, 13353 Berlin, Germany

**Keywords:** Prostate cancer, Radiation therapy, Postoperative, Salvage, Boost, Dose intensified

## Abstract

**Background:**

Postoperative adjuvant radiation therapy (ART) in T3 and R1 prostate cancer as well as salvage radiation therapy (SRT) in case of postoperative biochemical failure (BF) are established treatments. Dose-intensified postoperative radiation therapy (RT) schemes have shown superior biochemical control accompanied by increased toxicity rates. In our study we evaluate a novel risk adapted dose-intensified postoperative RT scheme.

**Methods:**

A consecutive series of prostate cancer patients receiving postoperative RT after radical prostatectomy using helical Tomotherapy between 04/2012 and 04/2015 was analyzed retrospectively. RT was administered using a simultaneous integrated boost (SIB) to the area at risk (37 fractions of 1.9 Gy, total dose: 70.3 Gy) being defined based on histopathological findings (T3/R1 region) and in few cases according to additional diagnostic imaging. The whole prostate bed was treated with a dose of 66.6 Gy (37 fractions of 1.8 Gy). Primary endpoints were acute and late genitourinary (GU) and gastrointestinal (GI) toxicities. Secondary endpoints included patient reported outcome as assessed by the International Prostate Symptom Score (IPSS), the International Consultation on Incontinence questionnaire (ICIQ) and prostate cancer specific Quality of Life questionnaire QLQ-PR25, as well as rates of BF.

**Results:**

A total of 69 patients were analyzed. Sixteen patients underwent ART and 53 patients SRT, respectively. The median follow-up was 20 months (range, 8–41 months). Seven (10.1%) and four (5.8%) patients experienced acute grade 2 GU and GI toxicity. Two patients (2.9%) had late grade 2 GU toxicity, whereas no late grade 2 GI nor any grade 3 acute or late GU or GI events were observed. When compared to the baseline IPSS scores (*p* = 1.0) and ICIQ scores (*p* = 0.87) were not significantly different at the end of follow-up. Patient reported Quality of life (QoL) showed also no significant difference. A total of seven patients (10.1%) experienced a biochemical recurrence with the 2-year biochemical progression-free survival (bPFS) being 91%.

**Conclusions:**

Postoperative RT for prostate cancer patients with a risk adapted dose-intensified SIB using helical tomotherapy is feasible and associated with favorable acute and late GU and GI toxicity rates, no significant change of IPSS-, ICIQ scores and patient reported QoL and results in promising bPFS rates.

## Background

Prostate cancer is the most common male cancer in developed countries and is the fifth most cause of death from cancer worldwide [1]. Radical prostatectomy (RP) offers good long-term control rates and survival in patients with cancer confined to the prostate [2]. However, 15 to 40% of patients develop biochemical failure (BF) after RP within 5 years [3–5]. In patients with high risk disease (extracapsular spread, seminal vesicle invasion or positive surgical margins) adjuvant radiation therapy (ART) improves biochemical progression-free survival (bPFS), overall survival (OS) and distant metastasis-free survival (DMFS) [6–9]. In the case of BF after RP, salvage radiation therapy (SRT) is the only potential curative treatment [10–12]. Moreover, dose-intensified radiation therapy (RT) showed a further improvement of biochemical relapse-free survival in the postoperative radiation setting [13–15]. Recently published data confirmed the benefit of dose escalation in SRT. Tendulkar et al. detected a significant reduction of BF after SRT with an applied dose ≥66 Gy vs. < 66Gy when analyzing a cohort of 2460 patients [16]. In addition, Stish et al. evaluated data of 1106 patients with SRT and registered a significantly reduced risk of BF when doses ≥68 Gy were used [17]. On the other hand, postoperative RT is also associated with genitourinary (GU) and gastrointestinal toxicity (GI), especially in dose-intensified radiation schemes. [13–15]. However, the increasing implementation of modern radiation techniques was reported to be associated with a decrease in toxicity, even in dose intensified radiation schemes [13, 18, 19]. Otherwise, the first report of the prospective randomized SAKK 09/10 trial showed a significantly increased patient reported genitourinary symptom burden in the dose intensified SRT arm (70 Gy) compared to the patients in the 64 Gy arm, irrespective of the applied radiation technique [20]**.** With the objective of combining the benefit of dose escalated RT with an assumed lower GU and GI toxicity rate using confined dose intensified radiation volumes, we used the helical tomotherapy for postoperative prostate cancer RT with a risk adapted dose-intensified simultaneous integrated boost (SIB).

## Methods

Between 04/2012 and 04/2015, 76 consecutive prostate cancer patients who received postoperative RT after RP were analyzed retrospectively. After exclusion of 7 patients due to either macroscopic lymphnode metastasis accompanied by a prostate-specific antigen (PSA) > 4 ng/ml (*n* = 5), bone metastasis (*n* = 1) or being lost to follow-up (*n* = 1) the remaining 69 patients were analyzed. Patients received ART [treatment 1–3 month after RP] (*n* = 16) or SRT [treatment >3 month after RP or persistent PSA after RP] (*n* = 53) using the tomotherapy treatment system. ART was performed in high risk patients with extracapsular spread, seminal vesicle invasion or positive surgical margins (pT3, R1). SRT was administered in patients with evidence of BF with two confirmed rises over a PSA value of 0.2 ng/ml or in patients with persistent PSA after RP, respectively. Furthermore, in some cases the SRT was indicated in patients with two consecutive rises of PSA with final PSA > 0.1 ng/ml or three consecutive rises according to the definition of BF in the SAKK 09/10 trial protocol [21]. Additional androgen deprivation therapy (ADT) was administered based on risk factors according to the discretion of the referring urologist.

Computed tomography (CT) based treatment planning was performed in supine position with comfortably filled bladder and empty rectum. Clinical target volume (CTV) P comprised the prostate bed, CTV S comprised the SIB-region and in the case of pelvic lymph node radiation a CTV L contained this area. The planning target volume (PTV) P (prostate bed) was defined as the CTV P plus 5 mm in all directions. PTV S (SIB-region) was defined as the CTV S plus 2 mm in all directions and PTV L contained CTV L with a margin of 5 mm. The posterior PTV P and PTV S margin differed from other directions with a width of 3 mm. For delineation of the SIB-volume the high risk region of the prostate bed was defined considering the histological reports of prostatectomy (R1 region, T3 region of infiltration or the tumor-bearing area). Due to pN1 status and patients need for security some patients also received radiation of pelvic lymph nodes.

A prostate bed dose (PTV P) of 66.6 Gy (37 fractions of 1.8 Gy, 7.4 weeks) and a SIB (PTV S) of 70.3 Gy (37 fraction of 1.9 Gy, 7.4 weeks) to the risk region of the prostate bed was administered in helical tomotherapy technique. A concurrent radiation of pelvic lymph nodes (PTV L) was applied with a dose of 45 Gy in 11 patients, 50.4 Gy in 4 patients and 54 Gy in one case.

For the delineation European Organization for Research and Treatment of Cancer (EORTC) guidelines were considered. As additional information in 8 cases a prostate specific membrane antigen gallium 68Ga labeled positron emission tomography/computed tomography (68Ga PSMA-PET/CT), in 2 cases a magnetic resonance imaging (MRI) and in 2 cases a choline positron emission tomography/computed tomography (choline PET/CT) was used.

The GU and GI toxicities were classified using the National Cancer Institute Common Terminology Criteria version 4.0 (CTCAEv4.0). Acute toxicity events were defined as symptoms during treatment and up to 3 months after the end of RT. After >3 month the symptoms were defined as late toxicities. Toxicity events were defined as symptoms increasing in grade over the respective baseline symptoms. Further monitoring of GU symptoms was performed using the International Prostate Symptom Score (IPSS) and the International Consultation on Incontinence questionnaire (ICIQ). The Quality of Life (QoL) was detected with the IPSS-QoL score and EORTC Quality of Life Questionnaire prostate cancer specific module PR25 (QLQ-PR25). The QLQ-PR25 module was used to measure symptom scales (urinary symptoms, bowel symptoms) and functional scales (sexual activity, sexual functioning). The GU and GI toxicities were assessed in three time periods using CTCAEv4.0 classification: baseline (before start of radiation therapy), acute (during and at end of RT) and late (>3 month after RT). IPSS, ICIQ, IPSS-Qol and QLQ-PR25 were assessed before beginning of RT and at the end of follow-up.

BF after completed RT was defined as any PSA exceeding 0.4 ng/ml and rising. In case of BF further diagnostics like 68Ga PSMA-PET/CT were applied to investigate the localization of recurrence.

The primary objective was to determine the rates of acute and late GU and GI toxicities. Secondary objectives were to document patient related outcomes as assessed by the IPSS and the ICIQ, measure patient reported QoL and to describe the rate of bPFS. Differences in IPSS sums, ICIQ sums and QLQ-PR25 scores between baseline and post-treatment were compared performing the students *t*-test. Actuarial bPFS rates were estimated using the Kaplan-Meier method. Time to event was calculated from the first day of treatment until biochemical recurrence or the last follow-up visit. Influence factors for bPFS were analyzed using the Cox-regression method. Two-sided *p* values <0.05 were considered statistically significant. The data were analyzed in SPSS (SPSS Inc., Chicago, IL, version 24.0).

The internal institutional review board approved a waiver for research authorization.

## Results

### Patient characteristics

The patient characteristics were summarized in Table 1 and the median follow-up was 20 month (range 8–41 months). Before RP patients had a median PSA level of 10 ng/ml. RT was delivered after a median of 10 months after RP (range, 1–155 months). PSA levels prior to RT ranged from 0 to 2.05 ng/ml with a median of 0.21 ng/ml. 23 patients (33.3%) were treated with an ADT. In the last follow up survey still 12 patients (17.4%) received an ADT. The ADT was given according to the discretion of the referring urologist. The 23 patients with ADT met one or more of the following treatment criteria: Either a PSA doubling time < 3 months, a symptomatic local disease, a pN1 status or a high PSA value > 0.7 ng/ml before start of RT. In 15 patients a concurrent radiation of pelvic lymph nodes due to pN1 status and in one case by reason of patients need for security was applied.

**Table 1:** Patient characteristics

Variable	(*N* = 69) *n* (%)
PSA before prostatectomy (ng/mL), median (range)	10.0 (0.8, 84.0)
Resection margins	
R0	31 (44.9%)
R1	38 (55.1%)
Gleason score	
≤ 7	40 (58.0%)
≥ 8	28 (40.6%)
missing	1 (1,4%)
Tumor classification	
pT2a	4 (5.8%)
pT2b	1 (1.4%)
pT2c	16 (23.2%)
pT3a	21 (30.4%)
pT3b	26 (37.7%)
pT4	1 (1.4%)
Lymphadenectomy performed	
No	9 (13.0%)
Yes	60 (87.0%)
Lymphnode classification	
N0	51 (73.9%)
N1	18 (26.1%)
Number of lymph nodes removed, median (range)	13.0 (1.0, 51.0)
Persistent PSA 4–12 weeks after prostatectomy	
< 0.1 ng/mL	46 (66.7%)
≥ 0.1 ng/mL	15 (21.7%)
< 0.5 ng/ml	54 (78.3%)
≥ 0.5 ng/ml	7 (10.1%)
missing	8 (11.6%)
PSA at start of RT	
< 0.5 ng/mL	57 (82.6%)
≥ 0.5 ng/mL	12 (17.4%)
Age at start of RT median (range) in years	66 (45, 78)
Time from surgery to RT start, median (range) in months	10.0 (1.0, 155.0)
ECOG performance status at treatment start	
0	12 (17.4%)
1	57 (82.6%)
RT technique	
Tomotherapy	69 (100%)
ADT during RT	
No	46 (66.7%)
Yes	23 (33.3%)
Pelvic nodal RT	
No	53 (76.8%)
Yes	16 (23.2%)

*Abbreviations*: *PSA* prostate specific antigen, *RT* radiation therapy, *ECOG* Eastern

Cooperative Oncology Group, *ADT* androgen deprivation therapy

### Acute toxicity

At the baseline (before onset of RT) 56 patients had grade 1, 8 patients had grade 2 GU and no patients suffered from GI toxicities. The acute toxicity was assessed during and at the end of postoperative RT. During treatment 7 patients (10.1%) experienced grade 2 GU and 4 patients (5.8%) grade 2 GI toxicity. In detail the acute GU toxicity was described as grade 2 dysuria in 3 patients and a grade 2 increase of urinary frequency in 4 patients. In all 3 cases of dysuria a complete remission was observed at the late toxicity follow-up, whereas one of these patients developed a late grade 2 change of urinary frequency. In addition, in the 4 reported cases of acute grade 2 variation of urinary frequency, symptoms alleviated to grade 1 urinary frequency level in the late follow-up. Furthermore, the 4 patients that suffered from grade 2 acute GI toxicity all had a transient grade 2 diarrhea with a reported remission in the late toxicity follow-up. No acute grade 3 or higher GU and GI toxicity was detected. Table 2 provides a detailed overview of acute GI and GU toxicity.

**Table 2:** Acute and late genitourinary and gastrointestinal toxicity

GU Toxicity	CTCAE highest grade^a^	During/End of RT (*N* = 69) *n* (%)	End of Follow-up (*N* = 69)^b^ *n* (%)
Dysuria	0	60 (87.0%)	65 (94.2%)
	1	6 (8.7%)	3 (4.3%)
	2	3 (4.3%)	0 (0.0%)
Hematuria	0	69 (100.0%)	66 (95.7%)
	1	0 (0.0%)	2 (2.9%)
Urinary frequency	0	59 (85.5%)	59 (85.5%)
	1	6 (8.7%)	8 (11.6%)
	2	4 (5.8%)	1 (1.4%)
Urinary incontinence	0	68 (98.6%)	57 (82.6%)
	1	1 (1.4%)	10 (14.5%)
	2	0 (0.0%)	1 (1.4%)
Urinary retention	0	67 (97.1%)	57 (82.6%)
	1	2 (2.9%)	11 (15.9%)
Urinary urgency	0	57 (82.6%)	61 (88.4%)
	1	12 (17.4%)	7 (10.1%)
Highest grade of GU symptoms	0	44 (63.8%)	34 (49.3%)
	1	18 (26.1%)	32 (46.6%)
	2	7 (10.1%)	2 (2.9%)
	3	0 (0.0%)	0 (0.0%)
GI Toxicity	CTCAE highest grade^a^	During/End of RT (*N*=69) *n* (%)	End of Follow-up (*N*=69) *n* (%)
Anal or rectal hemorrhage	0	67 (97.1%)	68 (98.6%)
	1	2 (2.9%)	1 (1.4%)
Diarrhea	0	45 (65.2%)	67 (97.1%)
	1	20 (29.0%)	2 (2.9%)
	2	4 (5.8%)	0 (0.0%)
Rectal pain	0	65 (94.2%)	69 (100%)
	1	4 (5.8%)	0 (0.0%)
Highest grade of GI symptoms	0	45 (65.2%)	66 (95.7%)
	1	20 (29.0%)	3 (4.3%)
	2	4 (5.8%)	0 (0.0%)
	3	0 (0.0%)	0 (0.0%)

*Abbreviations*: *GU* genitourinary, *GI* gastrointestinal, *CTCAE* Common Terminology Criteria of Adverse Events, *RT* radiation therapy

^a^toxicity events were defined as symptoms increasing in grade over the respective baseline symptoms

^b^one patient with no End of Follow up GU toxicity due to bladder resection in bladder cancer

### Late toxicity

Only 2 patients (2.9%) suffered from grade 2 late GU toxicity, whereas no late grade 2 GI or any other grade 3 or higher toxicity was documented. One patient with former acute grade 2 dysuria experienced a late grade 2 urinary frequency variation and one patient who reported grade 1 baseline and acute incontinence developed a late grade 2 incontinence. See Table 2 for detailed information of late GU and GI toxicity.

### IPSS, QoL and ICIQ

**Fig. 1:**
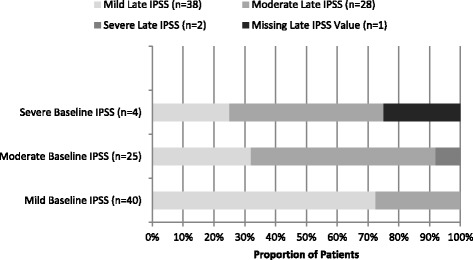
Late IPSS sum groups stratified by baseline IPSS sum groups. Abbreviations: IPSS = International Prostate Symptom Score

The evaluation of the IPSS of 69 patients showed a baseline IPSS sum with a mean of 7.7 (standard deviation (sd) of 6.2) and IPSS sum of 7.5 (sd 5.6) at the last late follow up after RT. No statistical significant difference of IPSS was detected (*p* = 1.0). The IPSS measures were depicted in Table 3. The comparison of baseline IPSS sum or sum group and last late IPSS sum or sum group values showed a considerable worsening (≥5 of IPSS) in 10 patients and a considerable improvement (≥5 of IPSS) in 7 patients [22, 23]. The subgroup of patients with a (≥5 of IPSS) aggravation showed in 8 cases a change from the mild to moderate IPSS symptoms group (mild 0–7, moderate 8–19, severe 20–35) and in 2 cases from the moderate to severe group. The patients who reported a significant IPSS improvement switched in four cases from moderate to mild IPSS sum group, in two cases from severe to moderate group and in one case from the severe to the mild sum group. Figure 1 depicts all IPSS group changes, including changes of IPSS sum groups without a sum difference of ≥5 IPSS.

**Table 3:** Baseline and late IPSS, ICIQ and IPSS-QoL assessment

Variable	Baseline		Last Follow-up		
	*n* (%)	mean (sd)	*n* (%)	mean (sd)	*p*-value^#^
IPSS:					
IPSS value	69 (100%)	7.7 (6.2)	68 (98.6%)	7.5 (5.6)	1.000
missing	0		1 (1.4%)		
IPSS grouped:					
Mild (0–7)	40 (58.0%)		38 (55.1%)		
Moderate (8–19)	25 (36.2%)		28 (40.6%)		
Severe (20–35)	4 (5.8%)		2 (2.9%)		
ICIQ:					
ICIQ value:	64 (92.8%)	5.8 (4.7)	68 (98.6%)	5.6 (4.2)	0.874
missing	5 (7.2%)		1 (1.4%)		
ICIQ grouped:					
No incontinence (0)	15 (21.7%)		15 (21.7%)		
Mild incontinence (1–5)	18 (26.1%)		20 (29.0%)		
Moderate incontinence (6–10)	21 (30.4%)		24 (34.8%)		
Severe incontinence (≥11)	10 (14.5%)		9 (13.0%)		
IPSS-QoL score grouped:^a^					
Satisfied (0–2)	47 (68.1%)		49 (71.0%)		
dissatisfied (3–6)	18 (26.1%)		19 (27.6%)		
missing	4 (5.8%)		1 (1.4%)		

*Abbrevations*: *sd* standard deviation, *IPSS* International Prostate Symptom Score, *ICIQ* International Consultation on Incontinence questionnaire, *QoL* quality of life

^#^by paired t-Test

^a^IPSS-QoL-Score: 0 = delighted to 6 = terrible

The patient reported QoL (IPSS-QoL score) was assessed before start of RT and at the end of follow-up. 64 patients reported their QoL using the IPSS-QoL score which is scaled from 0 = delighted to 6 = terrible. For analysis the score was dichotomized as 0–2 (satisfied) and 3–6 (dissatisfied) [22]. Overall 18 patients were dissatisfied before RT and 19 patients were dissatisfied after RT (Table 3). Further patient reported QoL measures were detected using the EORTC QQL-PR25 questionnaire. Patients reported no significant changes in urinary symptoms (*p* = 0.349), bowel symptoms (*p* = 0.888), sexual activity (*p* = 0.794) and sexual functioning (*p* = 1.000) at last follow compared to the baseline assessment. See Table 4 for details.

**Table 4:** Baseline and late patient reported quality of life scores (QLQ-PR25)

QLQ-PR25	Baseline		Last Follow-up		
	Number of respondents	mean (sd)	Number of respondents	mean (sd)	*p*-value^#^
Symptom Scales:^a^					
Urinary symptoms (PRURI)	41	26.9 (17.0)	68	25.1 (17.1)	0.349
Bowel symptoms (PRBOW)	40	5.8 (10.9)	67	5.6 (10.2)	0.888
Functional Scales:^b^					
Sexual activity (PRSAC)	34	57.4 (28.8)	51	57.2 (31.0)	0.794
Sexual functioning (PRSFU)	15	48.9 (15.1)	23	48.9 (14.7)	1.000

*Abbreviations*: *QLQ-PR25 EORTC* quality of life prostate cancer module PR25, *sd* standard deviation

^#^by paired t-Test

^a^Range 0–100, with a positive score indicating a worsening

^b^Range 0–100, with a positive score indicating an improvement

The ICIQ data of 64 patients showed an ICIQ sum with a mean of 5.8 (sd 4.7) before beginning and a mean of 5.6 (sd 4.2) at the last follow up. The distribution to the different ICIQ groups (0 = no incontinence, 1–5 = mild incontinence, 6–10 = moderate incontinence, ≥11 = severe incontinence) is depicted in Table 3. The comparison of ICIQ sum before and after RT showed no statistical significant worsening of ICIQ score after RT (*p* = 0.874).

### Biochemical control

Within a follow-up period of median 20 month 7 patients (10.1%) experienced BF. The time from RT to BF ranged from 4 to 40 month with a median of 16 month. To differ between local recurrence and distant failure further diagnostic was applied following the diagnosis of BF. In one patient the localization of recurrence was shown as bone metastasis in a bone scintigraphy. Further, in three patients a 68Ga PSMA-PET/CT scan detected a recurrence in the iliac lymph node region and the fifth patient suffered from a recurrence in the iliac and paraaortic lymph node region, also verified by a 68Ga PSMA-PET/CT scan. The other two patients with BF refused the further diagnostic procedures.

Thus, considering these findings, in 5 of the 7 patients with biochemical failure no local prostate bed recurrence was detected. In the other two cases there is no information available on the pattern of recurrence.

In our cohort a 2-year bPFS of 91% was observed. Additional analysis showed no significant factors for the development of a biochemical failure in multivariate cox regression. In univariate cox regression a persistent PSA ≥ 0.5 mg/ml after RP was associated with decreased bPFS (*p* = 0.046) and a presurgery PSA of >10 ng/ml showed a trend towards worsening of bPFS (*p* = 0.082, Table 5).

**Table 5:** Univariate and multiple Cox regression analysis of factors associated with biochemical recurrence-free survival

Factor	RR	CI	*p*
Univariate Cox regression:			
Age: ≤65 versus >65 (years)	1.004	0.224–4.495	0.995
ECOG performance status: 0 versus >1	0.898	0.105–7.692	0.922
Lymphnode involvement: N0 versus N1	0.476	0.057–3.956	0.492
Gleason score: ≤7 versus ≥8	2.224	0.496–9.976	0.297
Surgical margins: R0 versus R1	0.558	0.125–2.496	0.445
Androgen deprivation therapy: no or yes	0.91	0.175–4.741	0.911
Pelvic nodal RT: no or yes	0.635	0.076–5.288	0.674
Presurgery PSA: ≤10 versus >10 (ng/ml)	6.537	0.785–54.411	0.082
PSA Persistance after surgery: <0.5 versus ≥0.5 (ng/ml)	4.623	1.029–20.781	0.046
PSA at start of RT <0.5 versus ≥0.5 (ng/ml)	2.980	0.659–13.473	0.156
Multivariate Cox regression:			
Presurgery PSA: ≤10 versus >10 (ng/ml)	4.402	0.474–40.891	0.192
PSA Persistance after surgery: <0.5 versus ≥0.5 (ng/ml)	2.724	0.556–13.337	0.216

*Abbreviations*: *RR* relative risk, *CI* 95% confidence intervals, *RT* radiation therapy, *p p*-value, *ECOG* Eastern Cooperative Oncology Group, *PSA* prostate specific antigen

## Discussion

The results of this retrospective analysis in prostate cancer patients who received risk adapted dose-intensified RT after RP showed low rates of relevant acute and late GU and GI toxicity, no significant worsening of symptoms burden (IPSS, ICIQ), no significant change in QoL and comes along with promising biochemical control rates.

The reported minor rates of acute GU and GI toxicities, appeared during and at the end of RT, with 10.1% grade 2 GU toxicity and 5.8% grade 2 GI toxicity in absence of any grade 3 or higher acute toxicity were favorably comparable with and even were slightly below toxicity rates of already published postoperative RT trials. In addition, the low number of observed late toxicity with 2.9% of grade 2 GU toxicity and no other late ≥ grade 2 toxicity demonstrated a good tolerability of the administered dose-intensified postoperative RT. Several previous published retrospective studies showed remarkable toxicity rates associated with dose escalated RT. Administering a SRT (2D and 3D conformal techniques) with a median dose of 72 Gy Cozzarini et al. reported ≥ grade 2 late GU toxicity in 23.7 and 10% grade 3 late GU toxicity [CTCAEv3.0; median follow-up 99 month] [24]. Furthermore, Ost et al. observed 22% late ≥ grade 2 GU toxicity and 3% late grade 3 GU toxicity in patients who received a SRT in intensity-modulated radiation therapy (IMRT) technique with a median dose of 76 Gy [CTCAEv3.0; median follow-up 5 years] [13]. A comparison of three-dimensional (3D) versus IMRT SRT with doses between <66 Gy up to ≥70Gy, published by Goenka et al., showed a rate of ≥ grade 2 late GI toxicity in 1.9% of patients treated with IMRT versus 10.2% when treated with 3D conformal techniques. Aside from the 8.3% reduction of late GI toxicity using IMRT no significant difference in ≥ grade 2 late GU toxicity between both techniques was seen with an overall rate of 16.3% ≥ grade 2 late GU toxicity [CTCAEv3.0; median follow-up 60 month]. In the cohort treated with IMRT the IPSS was assessed and the average IPSS of patients was 5.24 (range 0–19) before SRT and the average maximum after SRT was 7 (range 0–30) [18, 25]. Whereas, the above mentioned studies all applied dose-intensified RT to the whole prostate bed, a trial published by Zilli et al. described the SRT (3D conformal technique and a minority treated with IMRT) with a boost to the suspected relapse regions visualized by aid of endorectal magnetic resonance imaging (eMRI) with 74 Gy and a prostate bed dose of 64 Gy. Acute Grade 2 GU and GI toxicities were reported in 12.3 and 19.3%. Furthermore, 1.8% of patients experienced acute grade 4 urinary obstruction and 6.4% of patients suffered from late grade 2 GU toxicity and grade 2 GI toxicity. Late Grade 3 GU toxicity was also observed in 6.4% and late grade 3 GI toxicity was reported in 1.8% [Radiation Therapy Oncology Group (RTOG) scoring and CTCAEv3.0] [26]. Our results show a lower rate of acute and late GU and GI toxicities, using a dose-intensified risk adapted boost of 70.3 Gy in a prostate bed partial volume and a dose of 66.6 Gy for the whole prostate bed. The smaller volume with a moderate intensified dose is assumed to cause the better tolerability. Moreover in our study all patients were treated with helical tomotherapy treatment technique, ensuring sufficient sparring of risk organs. However, concerning the late toxicity results it must be noted that the median follow-up of 20 month depicts a limitation of our study, because particularly late GU toxicities are even reported to occur up to 10 years after RT.

The to date only prospective dose intensified SRT trial (SAKK 09/10) recently reported comparable, but also slightly higher acute toxicity rates, using a similar intensified dose for radiation of the whole prostate bed. This trial observed 13% acute grade 2 GU toxicity and 0.6% grade 3 GU toxicity in the 64 Gy arm compared to 16.6% acute grade 2 toxicity and 1.7% grade 3 GU toxicity when 70 Gy were applied. Acute grade 2 GI toxicity occurred in 16%, grade 3 GI toxicity in 0.6% treated with 64 Gy compared to 15.4 and 2.3% acute grade 2 and 3 GI toxicity after 70 Gy, respectively [CTCAEv4.0]. No significant differences in acute toxicities between both arms were detected. Interestingly, the trial was also stratified for radiation technique (3D vs. IMRT/rotational RT) and in contrast to former mentioned studies no significant influence on acute toxicity was monitored. Furthermore, a significantly increased patient reported genitourinary symptom burden was observed in the 70 Gy arm [20].

In this context, it was assumed that high dose RT to urethra and vesico-urethral anastomosis as applied in dose escalated whole prostate bed RT schemes results in similar GU toxicity regardless of RT technique [27]. From that point of view the partial prostate bed SIB-volume could be supposed to explain the low GU toxicity rates in our study.

To discuss the toxicities it is also worth mentioning, that physician-assessed toxicity scoring systems like CTCAEv.4.0 may underestimate the patients symptoms burden and may neglect important problems of the patient [20, 28].

Therefore it is important to consider patient reported toxicity scoring systems like IPSS or ICIQ as well as patient reported QoL. In our study patients reported their urinary symptoms (IPSS questionnaire) and incontinence symptoms (ICIQ questionnaire) before the beginning of RT and at the end of follow-up. Comparing these surveys, both scores showed no statistical significant difference, neither worsening nor improvement (Table 3). Consequently, the patients’ evaluation of the treatment also showed a good long term tolerability of the applied postoperative radiation scheme. Comparable IPSS values where registered by Geonka et al. in dose intensified SRT using IMRT [18]. Another important evaluation of the treatment is the patients’ perception of their QoL. Patients assessment of their QoL before RT and at the end of follow-up showed no statistical significant difference. Thus, no long term variation of QoL could be detected (Tables 3 and 4).

Within a median follow-up of 20 month 7 patients (10.1%) developed a BF and the calculated 2-year bPFS was 91%. With the aid of further diagnostics (ga-68 PSMA-PET/CT and bone scintigraphy) in five cases a local recurrence in the prostate bed was excluded, confirming a promising local control. For the remaining two patients the localization of recurrence is unknown. These data of biochemical control represent a first hint that our applied postoperative radiation scheme seems to be a promising treatment option, whereas the short follow up period is a limitation. Further analysis of the data showed a persistent PSA ≥ 0.5 mg/ml after RP was significantly associated with decreased bPFS in the univariate analysis and a presurgery PSA >10 ng/ml showed also a trend for a decrease in bPFS: Whereas, further established risk factors showed no trend or significant influence in our cohort (Table 5). The limited follow-up and the size of the cohort are assumed to be reasons for these results.

However, it should be taken into account that approximately one-third of patients in our study received an additional ADT. Consequently it may have influenced the biochemical outcome as well as toxicity and QoL. For example, data of two randomized studies combining SRT and ADT were recently published. The GETUG-AFU 16 trial observed a significant improvement in 5-year progression-free survival for combination of 66 Gy SRT with additional 6 month goserelin versus 66 Gy SRT alone (80% versus 62%; *p* < 0.0001). No significant difference in overall survival (OS) was reported. In the combined treatment arm more acute < grade 3 toxicities were registered than in SRT alone [29]. RTOG 9601 trial applied either SRT with 64.8 Gy plus 24-month bicalutamide or SRT with 64.8 Gy alone. The authors reported a significant OS benefit for combined treatment after 12 years with 76.3% versus 71.3% (HR 0.77; 95% CI, 0.59–0.99; *p* = 0.04). Subgroup analysis showed particularly an OS benefit for patients with PSA levels of 0.7–1.5 ng/ml (HR 0.61; 95% CI, 0.39–0.95; *p* = 0.03) and PSA > 1.5 ng/ml (HR 0.45; 95% CI, 0.25–0.81; *p* = 0.007). Notable differences in toxicity between both arms were a rate of 70% gynecomastia in the bicalutamide group versus 11% in the SRT only group [30]. Nevertheless, it is important to bear in mind that ADT is associated with multiple short and long term side effects like bone loss, hot flashes, metabolic changes and gynecomastia [31]. Thus, considering these results and the reported toxicities it has not been finally clarified whether in postoperative treatment schemes like ours, all or neither patients should receive ADT, what kind of ADT should be applied and for which duration. Surely it remains an individual decision taking into account the patients risk factors.

As an additional limitation of our study, it must be kept in mind that the risk adapted SIB volume was, as described in the methods section, mainly defined considering histological findings and only in some cases additional diagnostic information (68Ga PSMA-PET/CT, MRI, choline-PET/CT) was applied. Consequently it is possible that in some cases the boost volume doesn’t reflect the whole high risk relapse region of the prostate bed and the postulated additional effect of the dose-intensified radiation would be missed. This limitation could be optimized by using additional information like 68Ga PSMA-PET/CT or MRI for the delineation procedure [32–35]. However, all patients received a dose of 66.6 Gy to the whole prostate bed and therefore a sufficient dose for potential control of the relapse region.

Taking into account the reported limitations (e.g. the short follow up) our presented treatment scheme with a risk adapted dose-intensified SIB using the tomotherapy treatment system and the reported low rate of toxicity, no variation of QoL and a favorable biochemical recurrence free outcome depicts an option for a modern well tolerated treatment in case of BF after RP or in high risk postoperative situations (T3, R1). However these retrospective findings should be verified in prospective trials. A future treatment scheme could be improved by using 68Ga PSMA-PET/CT and MRI additional to histological findings for definition of risk adapted SIB volumes in all treated patients. Otherwise, an early initiation of treatment (PSA <0.5 ng/ml) in the SRT setting should be considered in a future trial.

## Conclusions

With a low rate of relevant acute and late GU and GI toxicity, no significant worsening of symptoms burden (IPSS, ICIQ) and no significant change in QoL the postoperative prostate cancer RT with a dose-intensified risk adapted SIB applied in our study seems to be a favorable and well tolerable therapy that comes along with promising biochemical control rates. However as main limitations the short follow-up, the moderate size of the cohort and the retrospective design should be considered and thus our approach should be verified in a future prospective trial.

## Abbreviations

3D: Three-dimensional
68Ga PSMA-PET/CT: Prostate specific membrane antigen gallium 68Ga labeled positron emission tomography/computed tomography
ADT: Androgen deprivation therapy
ART: Adjuvant radiation therapy
BF: Biochemical failure
bPFS: Biochemical progression-free survival
choline PET/CT: Choline positron emission tomography/computed tomography
CT: Computed tomography
CTCAEv4.0: National Cancer Institute Common Terminology Criteria version 4.0
CTCAEv4.0: National Cancer Institute Common Terminology Criteria version 4.0
CTV: Clinical target volume
DMFS: Distant metastasis-free survival
eMRI: Endorectal magnetic resonance imaging
EORTC: European Organization for Research and Treatment of Cancer
GI: Gastrointestinal
GU: Genitourinary
ICIQ: International Consultation on Incontinence questionnaire
IMRT: Intensity-modulated radiation therapy
IPSS: International Prostate Symptom Score
MRI: Magnetic resonance imaging
OS: Overall survival
PSA: Prostate-specific antigen
PTV: Planning target volume
QLQ-PR25: EORTC Quality of Life Questionnaire prostate cancer specific module PR25
QoL: Quality of Life
RP: Radical prostatectomy
RT: Radiation therapy
RTOG: Radiation Therapy Oncology Group
SIB: Simultaneous integrated boost
SRT: Salvage radiation therapy
